# Supplementary material for: Cell metabolism regulates integrin mechanosensing via an SLC3A2-dependent sphingolipid biosynthesis pathway
Source: Nat Commun. 2018 Nov 19;9:4862. doi: 10.1038/s41467-018-07268-w (PMC6242995; doi:10.1038/s41467-018-07268-w)
Supplement: Supplementary file 1 — Supplementary Information [file 41467_2018_7268_MOESM1_ESM.pdf]

Supplementary Information

Cell metabolism regulates integrin mechanosensing via an SLC3A2-dependent sphingolipid biosynthesis pathway

Boulter et al.

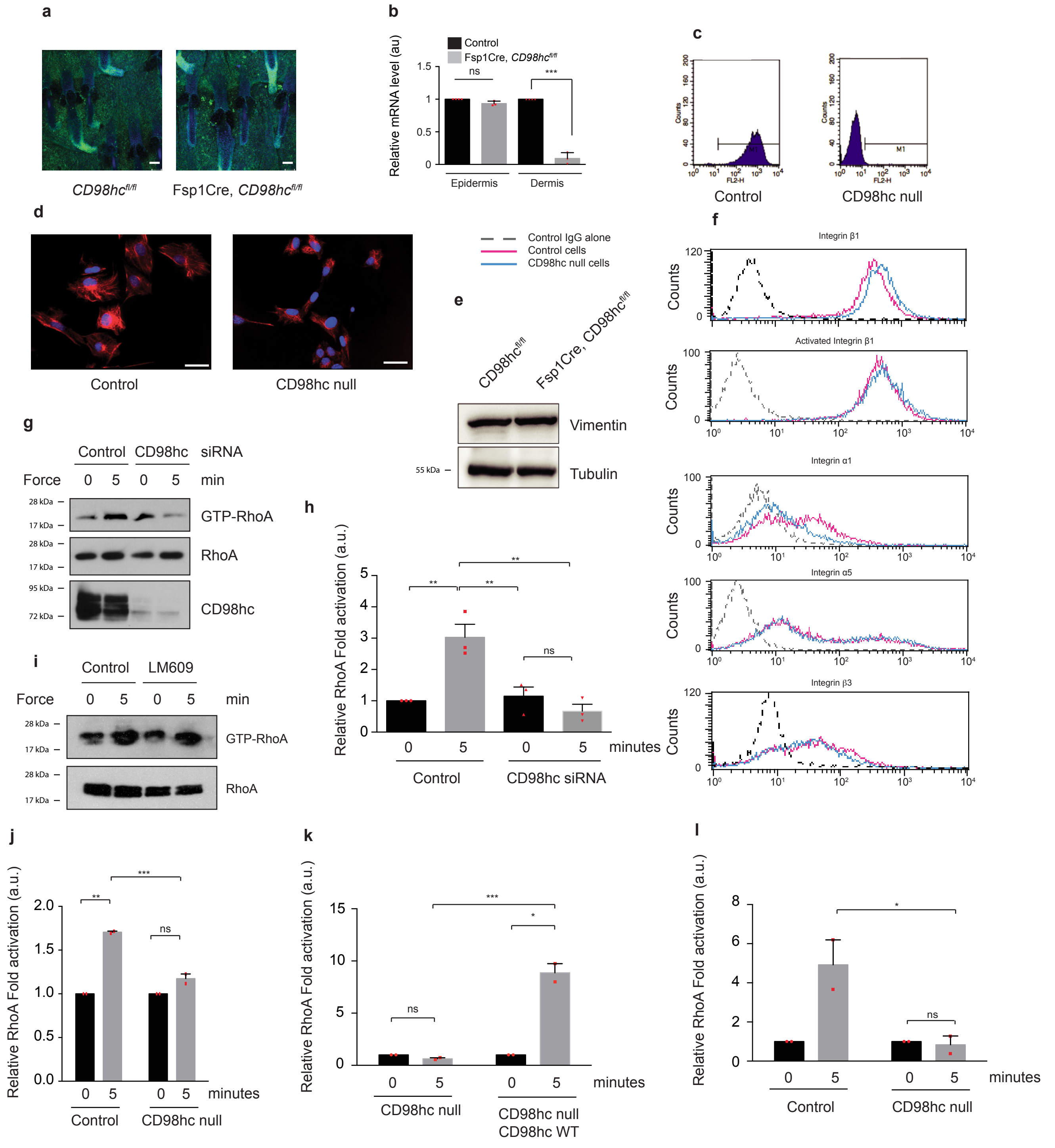

Supplementary Figure 1 - Characterization of CD98hc null fibroblasts

a Whole mounts of tail epidermis from CD98hcfl/fl or Fsp1Cre, CD98hcfl/fl mice labelled with antibodies against CD98hc(green) and counterstained with DAPI. Scale bar is 50µm. **b** Relative mRNA levels of CD98hc were measured by RT-qPCR on total mRNA extracted from epidermis or dermis of CD98hcfl/fl or Fsp1Cre, CD98hcfl/fl mice. n=3, mean with s.e.m. as error bars, ***P<0.001 in a Student's t-test. **c** CD98hc cell surface expression was measured by flow cytometry on control or CD98hc null dermal fibroblasts. **d, e** expression of the characteristic fibroblast marker vimentin was assayed by immunofluorescence (counterstain DAPI, scale bar is 50µm) (d) and western blotting on lysates of control or CD98hc null dermal fibroblasts (e). **f** cell surface expression of integrins α1, β3, β1, activated-β1 (9EG7) and α5 was measured by flow cytometry. **g** Activation of RhoA in CD98hc-depleted HeLa cells by siRNA upon application of mechanical forces on integrins. **h** Quantification of RhoA activation upon application of mechanical forces on integrins on CD98hc-depleted HeLa cells. Means are plotted with s.e.m. as error bars, (n=3 ** P<0.01 in a one-way ANOVA). **i** Mechanical-ly-coupled activation of RhoA is not altered by treatment with integrin β3 function-blocking antibody LM609 in HeLa cells. **j** Quantification of RhoA activation by G-LISA upon application of mechanical forces on integrins in control or CD98hc null cells. Means are plotted with s.e.m. as error bars (n=2 ** P<0.01, *** P<0.001 in a 2-way ANOVA). **k** Quantification of RhoA activation upon application of mechanical forces on integrins in CD98hc null cells or WT reconstituted CD98hc null cells. Signal was acquired with a Vilber Fusion Solo. Means are plotted with s.e.m. as error bars (n=2, * P<0.05, *** P<0.001 in a 2-way ANOVA). **l** Quantification of RhoA activation upon application of mechanical forces on integrins in control or CD98hc null cells using CollI-coated magnetic beads. Signal was acquired with a Vilber Fusion Solo. Means are plotted with s.e.m. as error bars (n=2, * P<0.05 in a 2-way ANOVA).

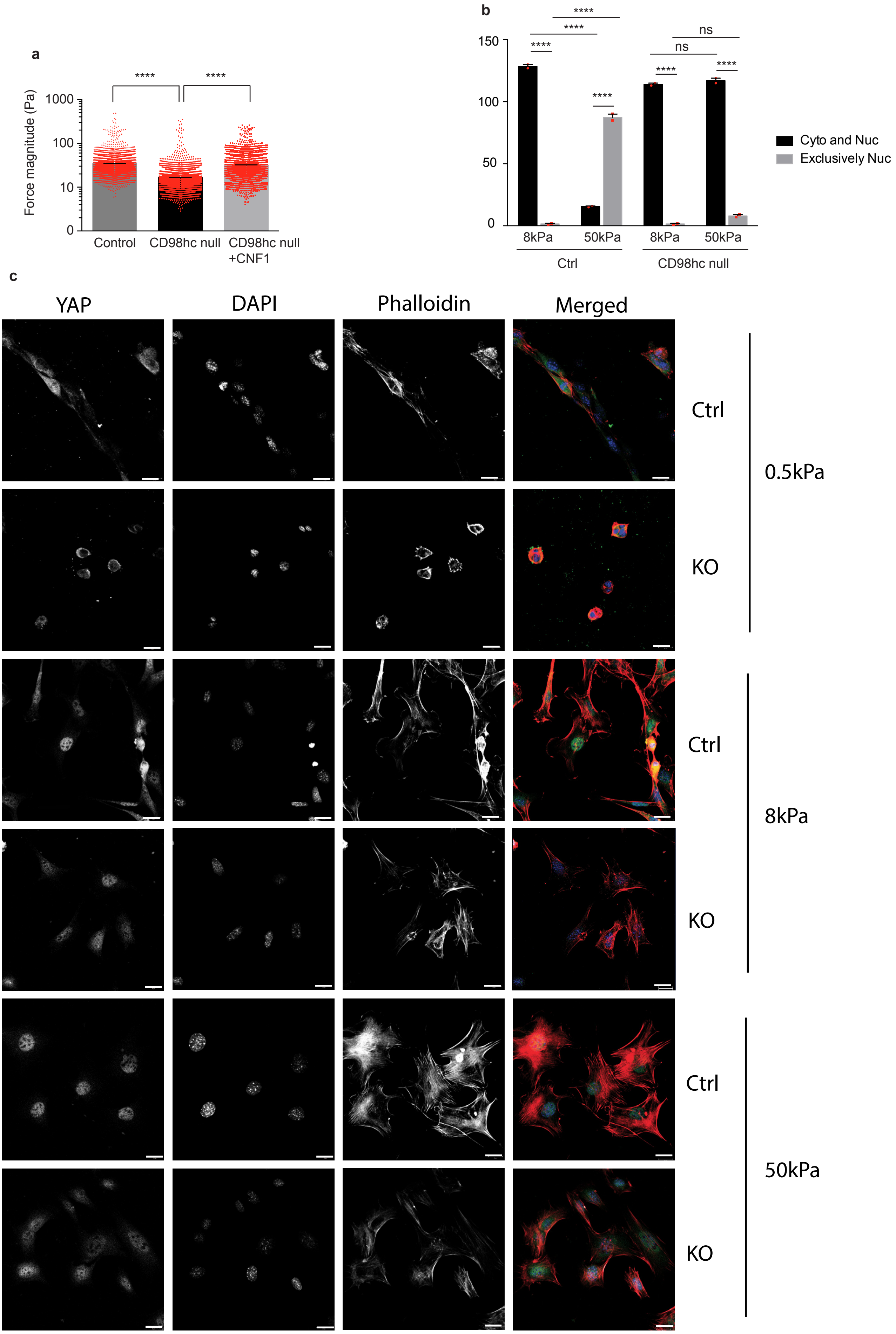

Supplementary Figure 2 – CD98hc depletion alters the mechanoregulation of YAP/Taz.
a Force intensities measured by traction force microscopy on CD98hcfl/fl (control) or Fsp1Cre, CD98hcfl/fl (CD98hc null) dermal fibroblasts grown 8kPa fibronectin-coated hydrogels for 24 hours and stimulated with CNF1 1µg/mL for 2 hours were individually plotted. At least 3844 force points per condition were measured at 10 different positions. n=2, means with s.e.m. as error bars, ****$P<0.0001$ in a Kruskal-Wallis test due to non-parametric distribution. **b** Immunofluorescence staining of YAP in control or CD98hc null cells plated on hydrogels of indicated stiffness. Scale bar is 20µm. **c** Quantification of the localization of YAP in the cytosol and nucleus (Cyto and Nuc) or nucleus exclusively (Exclusively Nuc) as observed by immunofluorescence. Means are plotted with s.e.m. as error bars from n=2. **** $P<0.0001$ in a 2-way ANOVA.

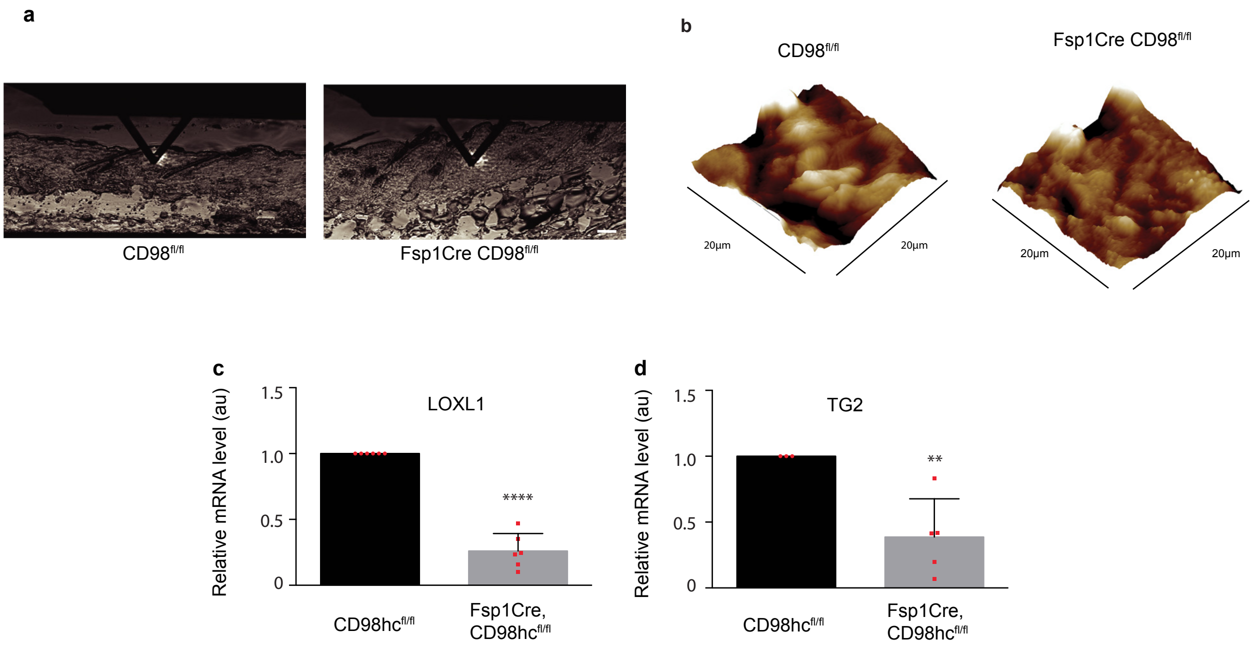

Supplementary Figure 3 – Genetic invalidation of CD98hc in the dermis affects its organization and architecture
a representative zone of the dermis scanned by AFM. **b** 3D reconstruction of the topographical images generated by scanning 20µm x 20µm surface areas of 10µm thick sagittal skin cryosections from CD98hcfl/fl or Fsp1Cre, CD98hcfl/fl mice using a pyramidal AFM probe by Peak Force QNM. Representative of n=4 mice in each condition. **c, d** Relative mRNA levels of LOXL1 and TG2 respectively, were measured by RT-qPCR on total mRNA extracted from skin of CD98hcfl/fl or Fsp1Cre, CD98hcfl/fl mice (mean with s.d. as error bars, n=6 **P<0.01, ****P<0.0001 in a Student's t-test).

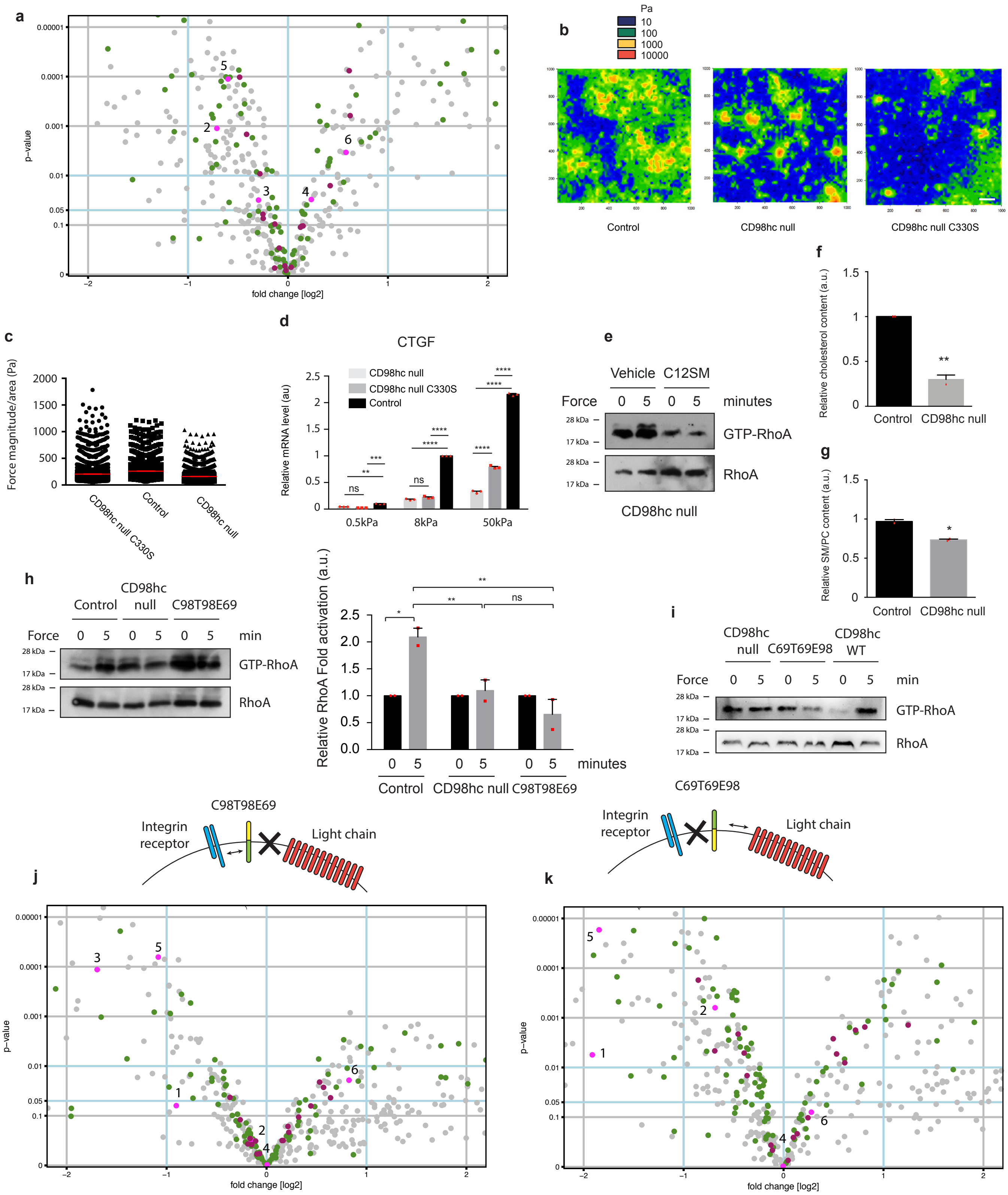

Supplementary Figure 4 – Characterization of C330S mutant and CD98hc-CD69 chimeras

a Loss of CD98hc affects lipid and amino acid metabolism. Volcano plot depicting metabolites fold changes versus p-value in CD98hc null versus CD98hc re-expressing CD98hc null cells. Pink, long chain based sphingoids, ceramides and phytoceramides; magenta, glycosphingolipids and sphingomyelin; green, amino acid metabolism. Numbered metabolites are analogous to the numbering in Figure 5I. 1, 3-ketosphinganine; 2, sphinganine; 3, N-palmitoyl-sphinganine; 4, N-palmitoyl-sphingosine; 5, palmitoyl dihydrosphingomyelin; 6, sphingosine. P-values were calculated using a Student's t-test, values are reported in Supplementary Data File. **b** Heatmaps of forces generated by control (left), CD98hc null (middle) or C330S-expressing CD98hc null dermal fibroblasts (right) grown on 8kPa FN-coated hydrogels for 24 hours. **c** Force intensities measured by traction force microscopy on aforementioned dermal fibroblasts grown 8kPa FN-coated hydrogels for 24 hours were individually plotted. At least 3844 force points per condition were measured at 10 different positions. Representative of two experiments. Red bars are means with s.e.m. as error bars. **d** Relative mRNA levels of CTGF was measured by RT-qPCR on total mRNA extracted from control, CD98hc null or C330S-expressing dermal fibroblasts grown on FN-coated hydrogels (means are plotted with s.e.m. as error bars, n=3, ** P<0.01, *** P<0.001, **** P<0.0001 in a 2way ANOVA). **e** Exogenous supply of C12 sphingomyelin does not restore mechanically-coupled RhoA activation in CD98hc null cells. **f** Loss of CD98hc induces a reduction of cellular cholesterol content. (mean +s.e.m., n=2, **P<0.01 in an unpaired Student's t-test). **g** Loss of CD98hc induces a reduction of cellular sphingomyelin content. Sphingomyelin (SM) content was normalized over Phosphatidyl choline (PC) content. (mean +s.e.m., n=2, *P<0.05 in an unpaired Student's t-test). **h, i** Re-expression of CD98hc-CD69 chimeras does not rescue mechanically-coupled RhoA activation. Right panel, quantification of RhoA activation upon re-expression of C98T98E69 and application of mechanical forces on integrins. means are plotted with s.e.m. as error bars from n=2 experiments, *P<0.05 **P<0.01 in a 2-way ANOVA. **j, k** Volcano plot depicting metabolites fold changes versus p-value in chimeras versus CD98hc expressing cells. Pink, long chain based sphingoids, ceramides and phytoceramides; magenta, glycosphingolipids and sphingomyelin; green, amino acid metabolism. Numbered metabolites are analogous to the numbering in Figure 5I. 1, 3-ketosphinganine; 2, sphinganine; 3, N-palmitoyl-sphinganine; 4, N-palmitoyl-sphingosine; 5, palmitoyl dihydrosphingomyelin; 6, sphingosine. P-values were calculated using a Student's t-test, values are reported in Supplementary Data File.

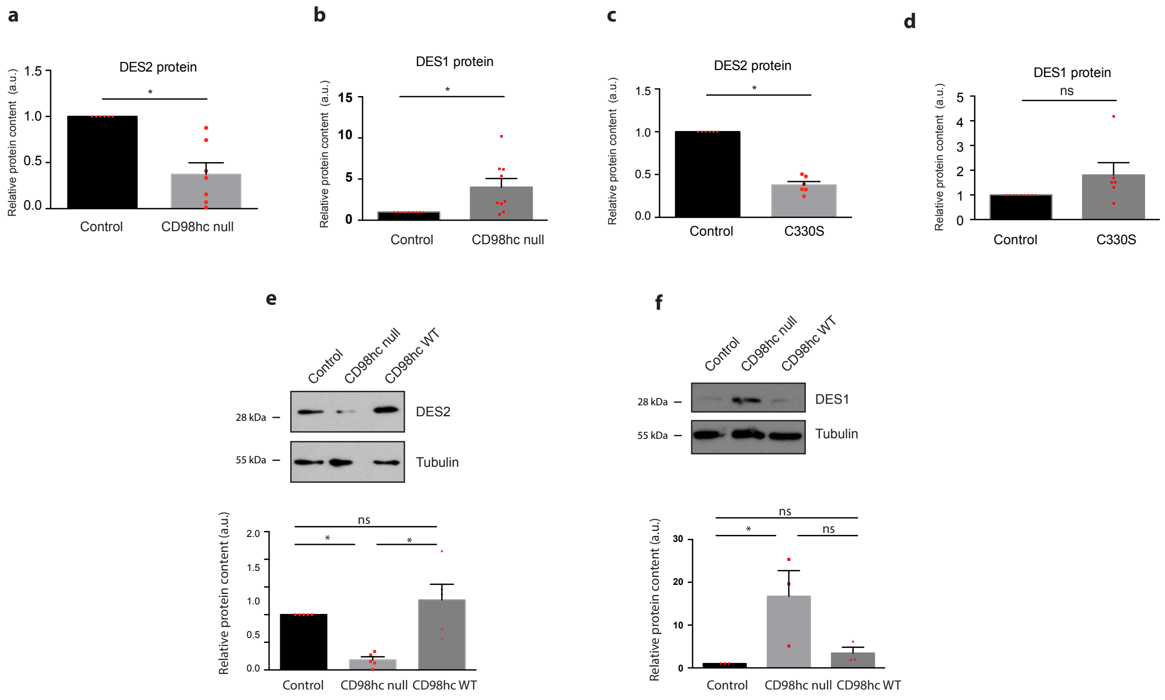

Supplementary Figure 5 – Re-expression of WT CD98hc in CD98hc null cells rescues the levels of DES1 and DES2
a, b, c and **d** Loss of CD98hc reduces the expression of DES2 and induces DES1. means are plotted from respectively n=7 or n=9 experiments. *P<0.05 in a Wilcoxon test. **e, f** Re-expression of WT CD98hc in CD98hc null cells rescues the levels of DES2 and DES1 respectively. Means are plotted with s.e.m. as error bars from n=3 experiments, ns P>0.05 *P<0.05 in a Kruskall-Wallis test.

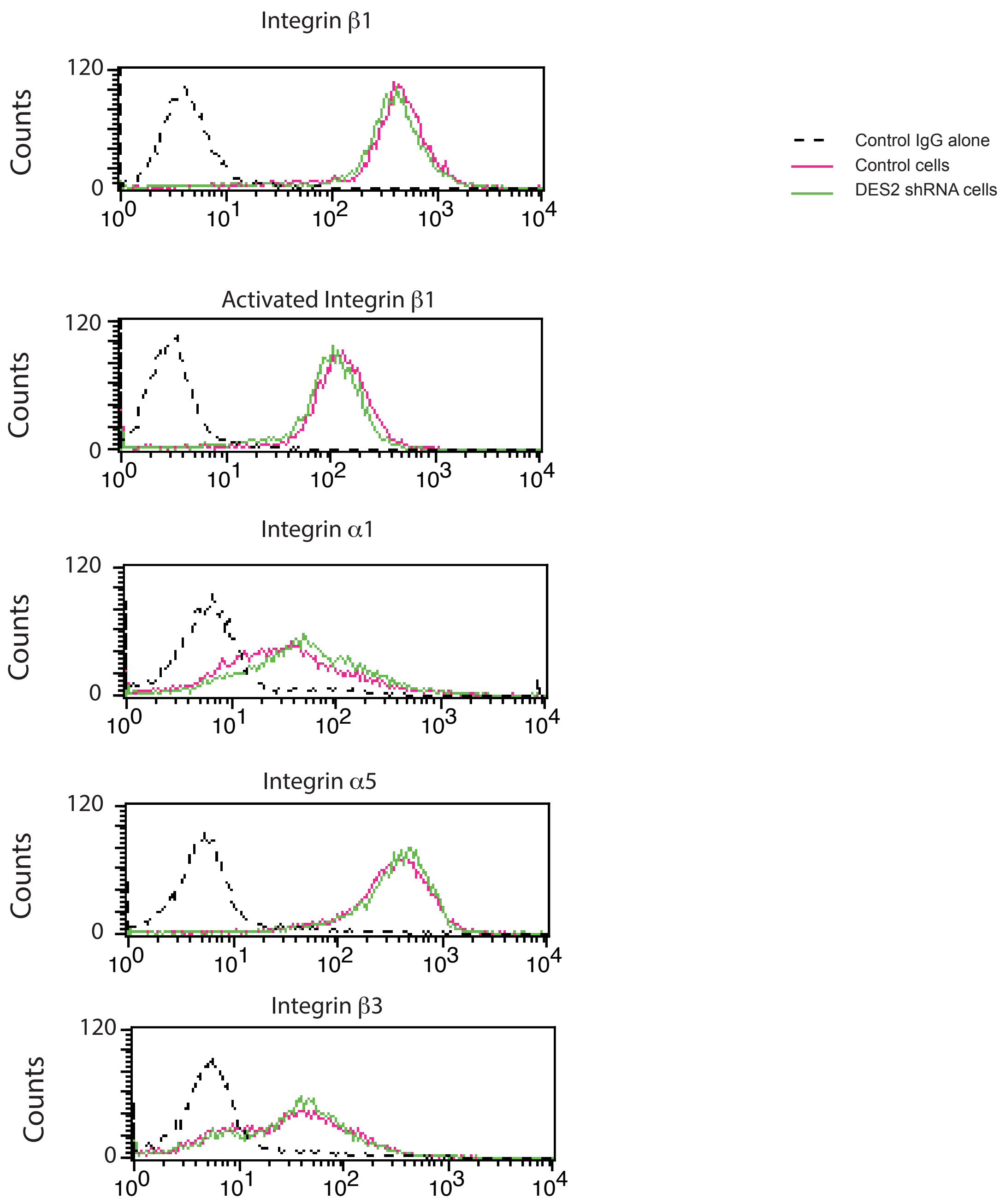

Supplementary Figure 6 – Cell surface expression of integrins is not affected by DES2 depletion.
Cell surface expression of integrins α1, β3, β1, activated-β1 (9EG7) and α5 was measured by flow cytometry.

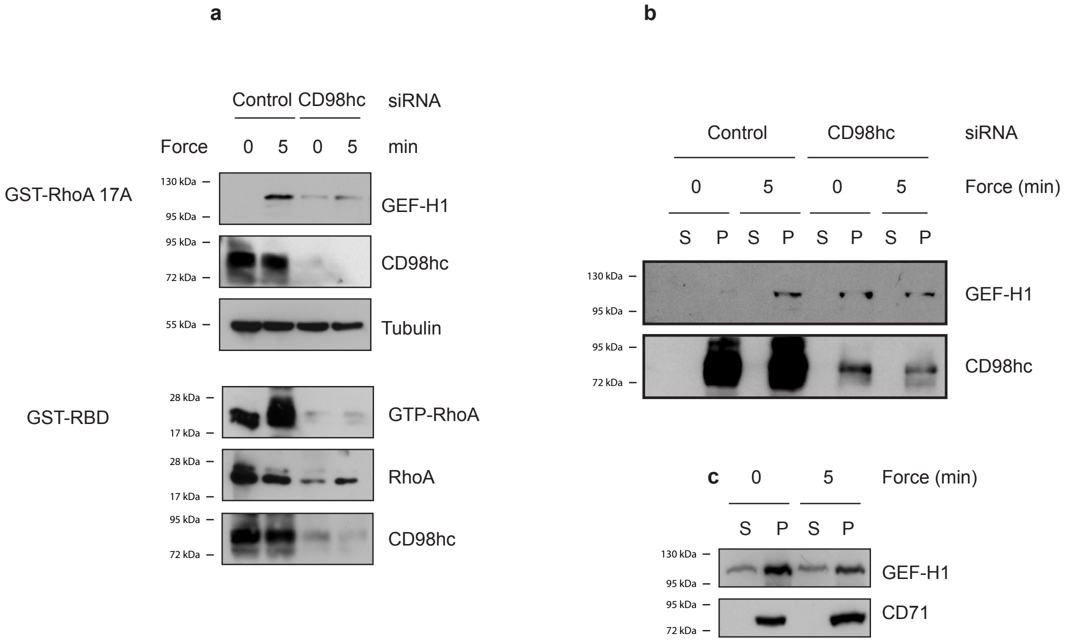

Supplementary Figure 7 – Loss of CD98hc in Hela cells induces the same effects as in dermal fibroblasts.
a HeLa cells were transfected with control or CD98hc siRNA for 72 h. Active RhoA or active RhoGEFs were pulled-down from cell lysates with GST-RBD or GST-RhoA17A beads respectively, following stimulation with FN-coated magnetic beads and a permanent magnet. Bound proteins and total cell lysates were resolved by SDS-PAGE and analysed by Western blotting. **b** HeLa cells were transfected with control or CD98hc siRNA for 72 h. Cells were stimulated with fibronectin-coated magnetic beads. Cells were lysed and lysates were fractionated into cytosolic (s) and membrane (p) fractions by centrifugation. Total cell lysates were resolved by SDS-PAGE and analyzed by Western blotting. **c** Membrane localization of GEF-H1 in CD98hc null fibroblasts expressing the C330S mutant.

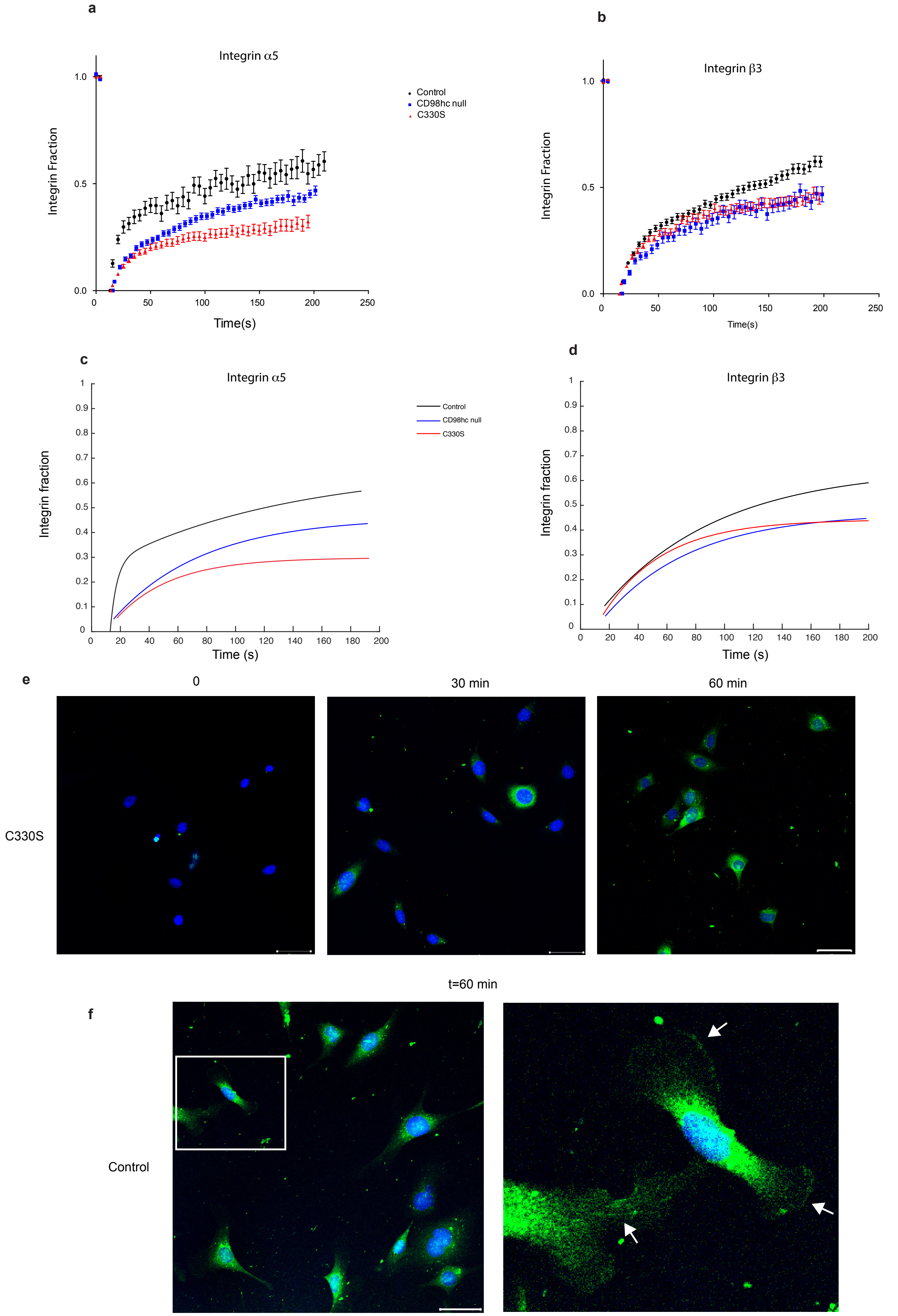

Supplementary Figure 8 – Integrin FRAP data processing and integrin trafficking
a, b Fluorescence recovery after photobleaching over time of integrin α5 or β3 in control (black), CD98hc null (blue) or C330S (red) expressing cells. Means are plotted with s.e.m as error bars. **c, d** Curve fits of FRAP data as calculated by curve fitting of FRAP data. R2 = 0.9736, 0.9838 and 0.9741 respectively for control, CD98hc null and C330S cells for integrin α5. R2=0.9701, 0.9735 and 0.9797 respectively for control, CD98hc null and C330S cells for integrin β3 . **e** trafficking of integrin β1 in C330S cells. Integrin β1 was labeled with Alexa 488 coupled antibody then integrin trafficking was chased for indicated time. Extracellular staining was quenched and only intracellular labeled integrin is observed. scale bar is 50μm. **f** Crop in image of integrin β1 trafficking showing recycling of integrin β1 in membranes and adhesion complexes (white arrows), scale bar is 50μm.

a

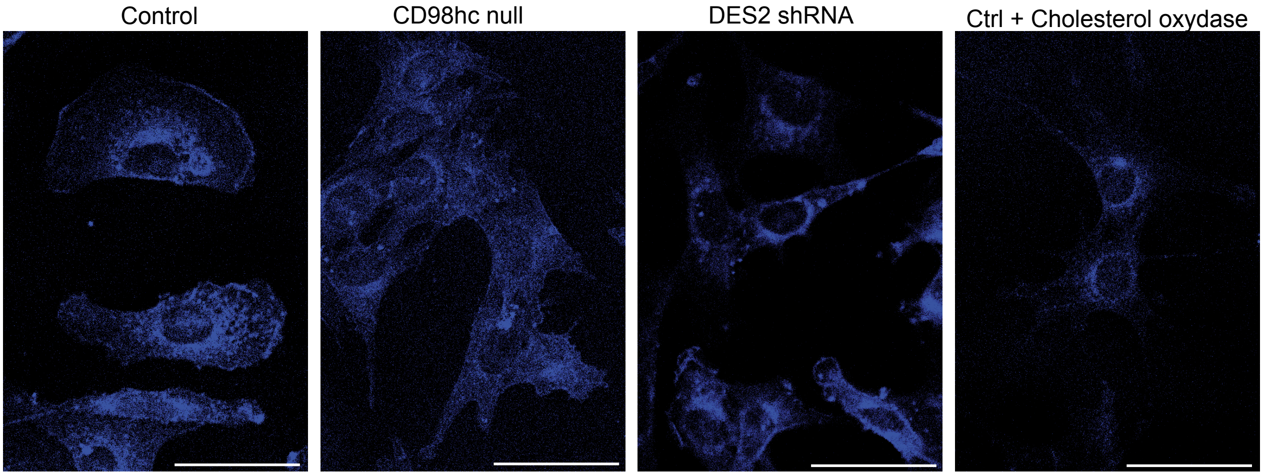

b

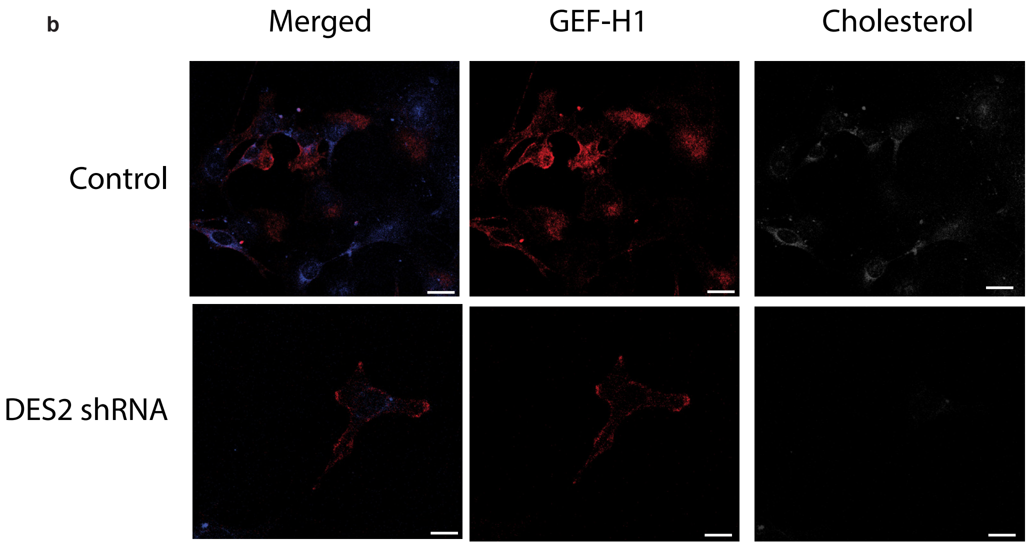

Supplementary Figure 9 – Loss of CD98hc or DES2 affects cholesterol staining
a Immunofluorescence staining of cholesterol using filipin III in control, CD98hc null, DES2 shRNA and cholesterol oxidase-treated control cells. Scale bar is 50µm. **b** Immunofluorescence staining of GEF-H1 and cholesterol using filipin III in control and DES2 shRNA cells. Scale bar is 50µm.

	WT/ CD98hc	CD98hc_null/ CD98hc	C330S/ CD98hc	C109S/ CD98hc	C98T98E69/ CD98hc	C69T69E98/ CD98hc	sub_pathway
1,2-dilinoleoyl-GPC (18:2/18:2)	1,0	0,9	0,9	0,7	1,7	1,2	Phospholipid Metabolism
1,2-dioleoyl-GPC (18:1/18:1)*	0,7	0,8	1,1	0,8	0,8	0,8	Phospholipid Metabolism
1,2-dioleoyl-GPE (18:1/18:1)	1,2	1,6	1,0	2,1	1,3	1,1	Phospholipid Metabolism
1,2-dioleoyl-GPG (18:1/18:1)	1,1	1,0	0,6	0,6	0,5	0,2	Phospholipid Metabolism
1,2-dioleoyl-GPS (18:1/18:1)	0,6	0,9	1,0	1,1	0,9	0,9	Phospholipid Metabolism
1,2-dipalmitoyl-GPC (16:0/16:0)	0,8	1,1	1,1	2,4	1,4	1,5	Phospholipid Metabolism
1,2-distearoyl-GPC (18:0/18:0)	0,7	0,6	1,3	1,0	1,0	1,0	Phospholipid Metabolism
1,2-distearoyl-GPG (18:0/18:0)	1,1	1,4	1,2	2,8	1,6	1,4	Phospholipid Metabolism
1-linoleoyl-2-arachidonoyl-GPC (18:2/20:4n6)*	1,5	1,4	1,0	4,1	2,3	2,9	Phospholipid Metabolism
1-oleoyl-2-arachidonoyl-GPC (18:1/20:4)*	1,1	1,6	1,2	4,4	1,9	2,5	Phospholipid Metabolism
1-oleoyl-2-linoleoyl-GPC (18:1/18:2)*	0,6	0,7	1,0	0,5	1,1	0,8	Phospholipid Metabolism
1-oleoyl-2-linoleoyl-GPE (18:1/18:2)*	1,0	0,7	0,9	0,4	0,9	0,6	Phospholipid Metabolism
1-palmitoleoyl-2-linoleoyl-GPC (16:1/18:2)*	0,9	1,1	0,9	1,4	1,4	1,1	Phospholipid Metabolism
1-palmitoleoyl-2-oleoyl-GPC (16:1/18:1)*	1,0	1,4	0,8	1,7	1,4	0,9	Phospholipid Metabolism
1-palmitoyl-2-arachidonoyl-GPC (16:0/20:4)	1,2	1,6	1,1	6,9	1,8	2,7	Phospholipid Metabolism
1-palmitoyl-2-arachidonoyl-GPE (16:0/20:4)*	1,0	1,3	1,5	4,9	1,9	2,2	Phospholipid Metabolism
1-palmitoyl-2-linoleoyl-GPC (16:0/18:2)	0,6	0,7	1,1	0,7	0,9	0,8	Phospholipid Metabolism
1-palmitoyl-2-linoleoyl-GPE (16:0/18:2)	0,9	0,8	1,0	0,9	1,0	0,8	Phospholipid Metabolism
1-palmitoyl-2-linoleoyl-GPS (16:0/18:2)	0,9	0,8	1,1	0,8	1,3	1,0	Phospholipid Metabolism
1-palmitoyl-2-oleoyl-GPC (16:0/18:1)	0,7	0,8	1,1	1,0	0,9	0,8	Phospholipid Metabolism
1-palmitoyl-2-oleoyl-GPE (16:0/18:1)	0,9	1,0	1,1	1,5	1,0	0,9	Phospholipid Metabolism
1-palmitoyl-2-oleoyl-GPG (16:0/18:1)	1,3	1,3	1,4	2,5	1,7	1,1	Phospholipid Metabolism
1-palmitoyl-2-oleoyl-GPS (16:0/18:1)	0,5	0,6	1,3	0,8	0,6	0,5	Phospholipid Metabolism
1-palmitoyl-2-palmitoleoyl-GPC (16:0/16:1)*	0,7	0,9	1,0	1,2	0,8	0,9	Phospholipid Metabolism
1-palmitoyl-2-stearoyl-GPC (16:0/18:0)	0,8	0,7	1,3	1,1	1,1	1,1	Phospholipid Metabolism
1-stearoyl-2-arachidonoyl-GPC (18:0/20:4)	1,3	1,5	1,2	6,5	2,0	3,4	Phospholipid Metabolism
1-stearoyl-2-arachidonoyl-GPE (18:0/20:4)	0,7	1,1	1,4	2,6	1,8	2,1	Phospholipid Metabolism
1-stearoyl-2-arachidonoyl-GPI (18:0/20:4)	1,0	1,2	1,2	2,8	1,7	1,5	Phospholipid Metabolism
1-stearoyl-2-linoleoyl-GPC (18:0/18:2)*	0,8	0,7	0,9	0,5	0,8	0,7	Phospholipid Metabolism
1-stearoyl-2-linoleoyl-GPE (18:0/18:2)*	1,0	0,8	1,0	0,5	1,1	0,8	Phospholipid Metabolism
1-stearoyl-2-linoleoyl-GPS (18:0/18:2)	0,8	0,6	1,1	0,7	1,2	1,1	Phospholipid Metabolism
1-stearoyl-2-oleoyl-GPC (18:0/18:1)	0,6	0,6	1,2	0,5	0,5	0,5	Phospholipid Metabolism
1-stearoyl-2-oleoyl-GPE (18:0/18:1)	0,9	1,0	1,0	1,2	1,0	0,9	Phospholipid Metabolism
1-stearoyl-2-oleoyl-GPG (18:0/18:1)	1,4	0,7	1,0	0,9	0,9	0,6	Phospholipid Metabolism
choline	0,7	1,3	0,9	0,9	0,7	0,6	Phospholipid Metabolism
choline phosphate	0,6	0,8	1,0	1,0	1,2	1,1	Phospholipid Metabolism
cytidine 5'-diphosphocholine	0,8	0,7	1,1	0,7	0,8	0,7	Phospholipid Metabolism
cytidine-5'-diphosphoethanolamine	0,5	1,6	0,6	1,1	0,8	0,5	Phospholipid Metabolism
glycerophosphoethanolamine	0,8	0,7	0,9	0,2	0,2	0,1	Phospholipid Metabolism
glycerophosphoinositol*	0,5	0,4	1,4	0,5	0,4	0,2	Phospholipid Metabolism
glycerophosphorylcholine (GPC)	0,9	0,7	1,0	0,5	0,3	0,2	Phospholipid Metabolism
3-ketosphinganine	0,6	0,0	3,4	0,0	0,5	0,3	Sphingolipid Metabolism
glycosyl-N-palmitoyl-sphingosine	1,4	0,7	0,7	1,4	1,2	1,1	Sphingolipid Metabolism
glycosyl-N-stearoyl-sphingosine	1,6	0,8	0,5	1,2	0,9	0,7	Sphingolipid Metabolism
lactosyl-N-palmitoyl-sphingosine	2,5	1,5	0,9	1,1	0,8	0,8	Sphingolipid Metabolism
N-palmitoyl-sphinganine (d18:0/16:0)	1,0	0,8	2,8	0,5	0,3	0,2	Sphingolipid Metabolism
N-palmitoyl-sphingosine (d18:1/16:0)	1,1	1,2	0,9	1,6	1,0	1,0	Sphingolipid Metabolism
palmitoyl dihydrosphingomyelin (d18:0/16:0)*	0,8	0,7	1,1	0,6	0,5	0,3	Sphingolipid Metabolism
palmitoyl sphingomyelin (d18:1/16:0)	0,8	0,8	0,9	1,0	0,9	0,9	Sphingolipid Metabolism
sphinganine	1,4	0,6	2,7	0,8	0,9	0,6	Sphingolipid Metabolism
sphingomyelin (d18:1/14:0, d16:1/16:0)*	0,8	0,9	0,9	1,1	1,1	1,2	Sphingolipid Metabolism
sphingomyelin (d18:1/15:0, d16:1/17:0)*	0,8	0,9	0,9	1,1	1,1	1,1	Sphingolipid Metabolism
sphingomyelin (d18:1/17:0, d17:1/18:0, d19:1/16:0)	1,3	0,8	0,8	1,1	1,4	1,4	Sphingolipid Metabolism
sphingomyelin (d18:1/18:1, d18:2/18:0)	0,7	1,0	0,7	1,4	1,0	1,0	Sphingolipid Metabolism
sphingomyelin (d18:1/20:0, d16:1/22:0)*	0,8	1,1	0,5	1,6	0,8	0,6	Sphingolipid Metabolism
sphingomyelin (d18:1/20:1, d18:2/20:0)*	0,6	1,5	0,4	3,1	0,9	0,6	Sphingolipid Metabolism
sphingomyelin (d18:1/22:1, d18:2/22:0, d16:1/24:1)*	0,9	1,1	1,0	1,9	1,2	1,4	Sphingolipid Metabolism
sphingomyelin (d18:1/24:1, d18:2/24:0)*	1,0	0,8	1,0	1,2	0,9	0,9	Sphingolipid Metabolism
sphingomyelin (d18:2/14:0, d18:1/14:1)*	1,2	1,3	0,8	2,6	1,6	2,4	Sphingolipid Metabolism
sphingomyelin (d18:2/16:0, d18:1/16:1)*	0,7	1,0	0,8	1,5	1,2	1,5	Sphingolipid Metabolism
sphingomyelin (d18:2/23:0, d18:1/23:1, d17:1/24:1)*	0,6	0,9	1,0	2,1	1,4	1,8	Sphingolipid Metabolism
sphingomyelin (d18:2/24:1, d18:1/24:2)*	1,1	1,1	0,8	2,0	1,5	1,6	Sphingolipid Metabolism
sphingosine	2,5	1,5	0,9	2,2	1,8	1,2	Sphingolipid Metabolism
stearoyl sphingomyelin (d18:1/18:0)	1,1	1,0	0,8	1,4	0,9	0,8	Sphingolipid Metabolism

Supplementary Table 1 – Effect of the expression of CD98hc constructs on sphingolipid and phospholipid levels.

The table displays the fold change of each metabolite between control, CD98ch null, C330S, C109S, C98T98E69 or C69T69E98, and WT CD98hc expressing CD98hc null cells. n=4. Fold changes are indicated with student's t-test comparison color coded as follow, green, downregulation with P<0.01; light blue, downregulation with P<0.05; purple, upregulation with P<0.01; pink, upregulation with P<0.05; blank, P>0.05.

CD98hc variant	Integrin signaling	Aminoacid transport	Reference
CD98hc	+	+	[1,2]
C109S	+	-	[1]
C330S	+	+	[1]
C98T98E69	+	-	[2]
C69T69E98	-	+	[2]

Supplementary Table 2 – Summary of the functional features of mutants and chimeras used in this study.

Supplementary information - original blots

1c

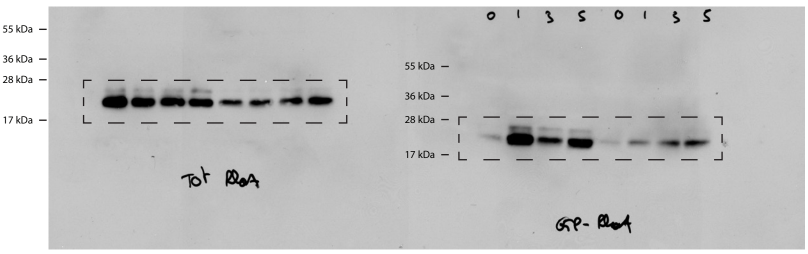

1d

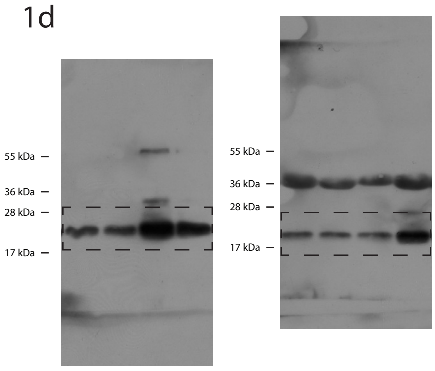

1e

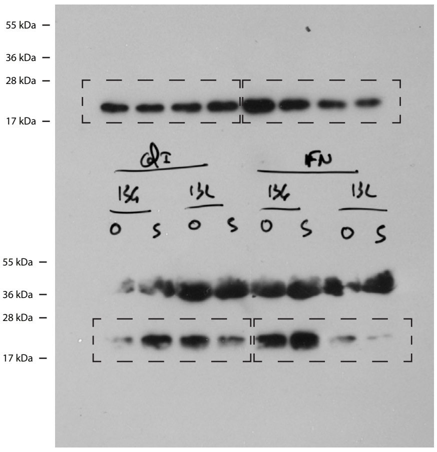

1f

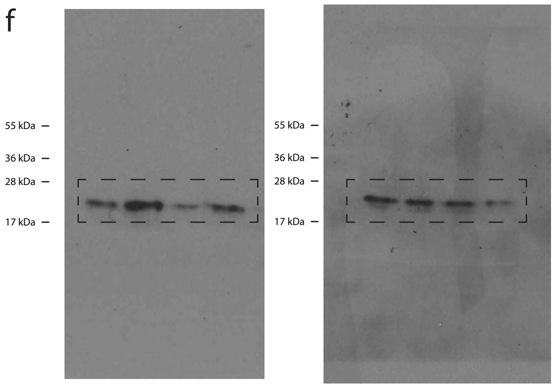

1g

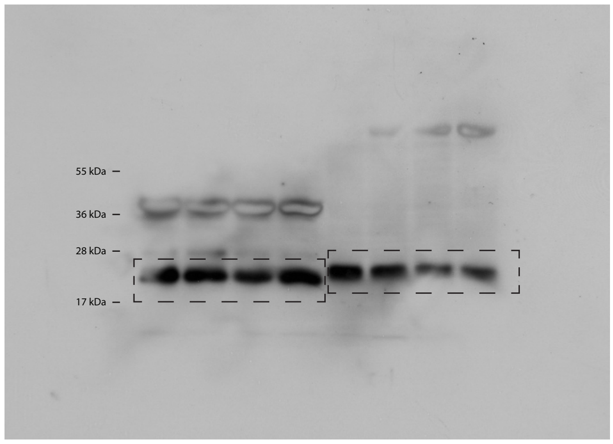

1h

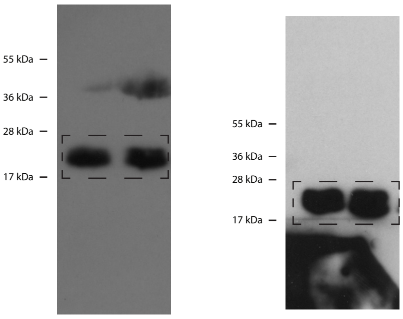

1j

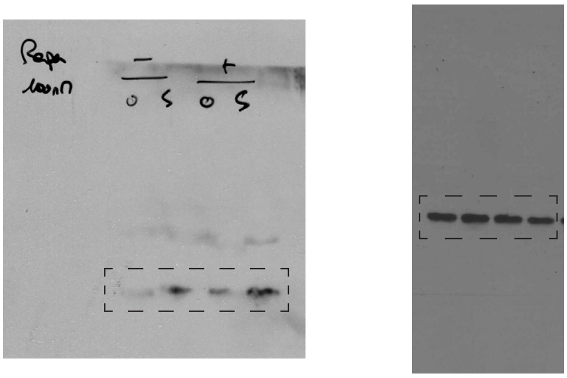

Supplementary information - original blots

2b

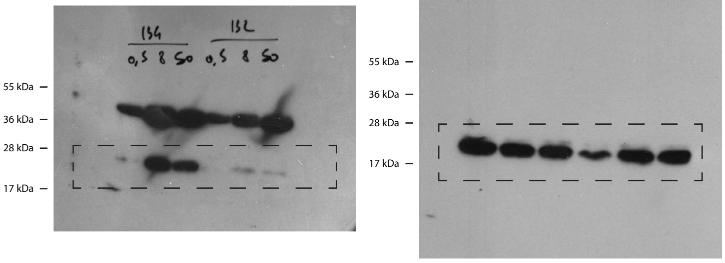

3j

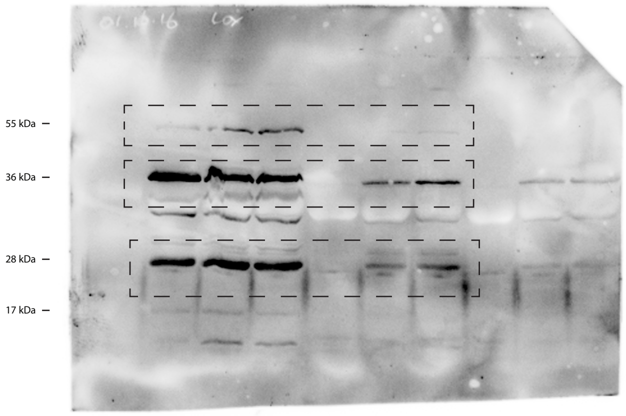

4b

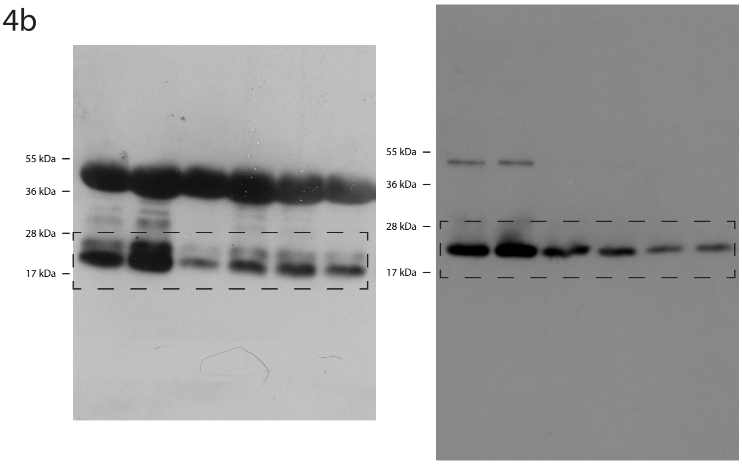

4e

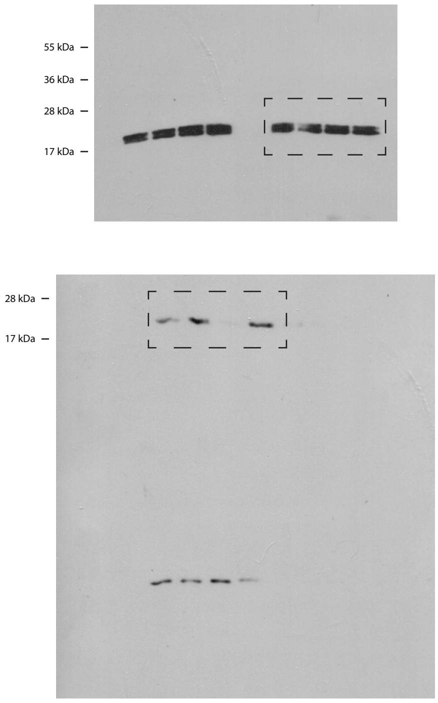

4f

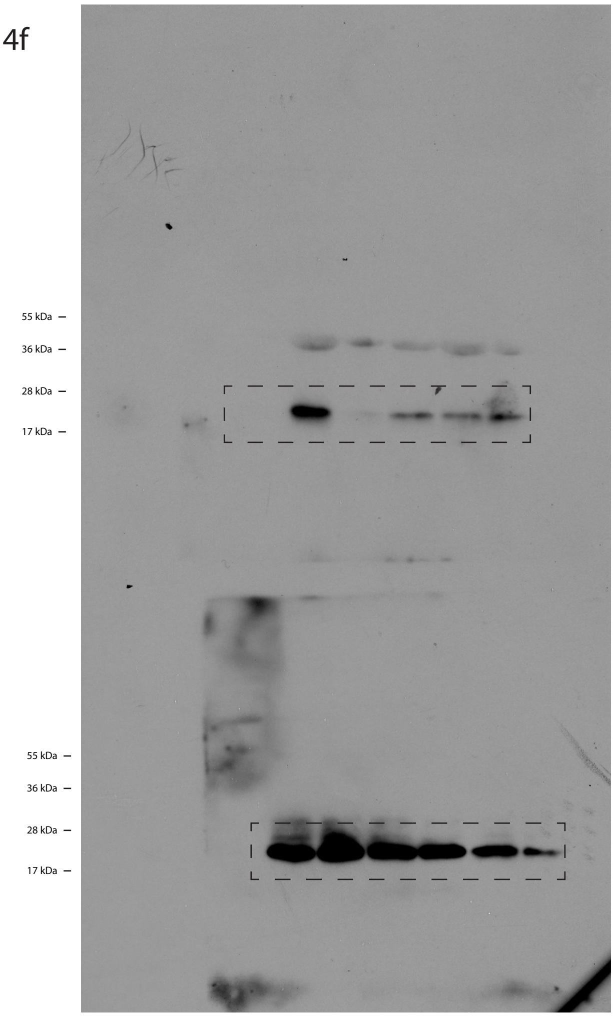

4g

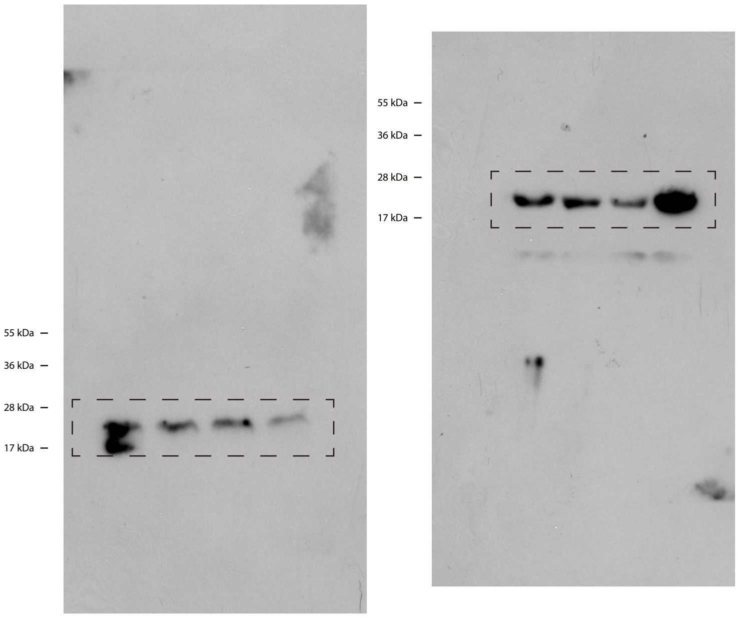

Supplementary information - original blots

5a

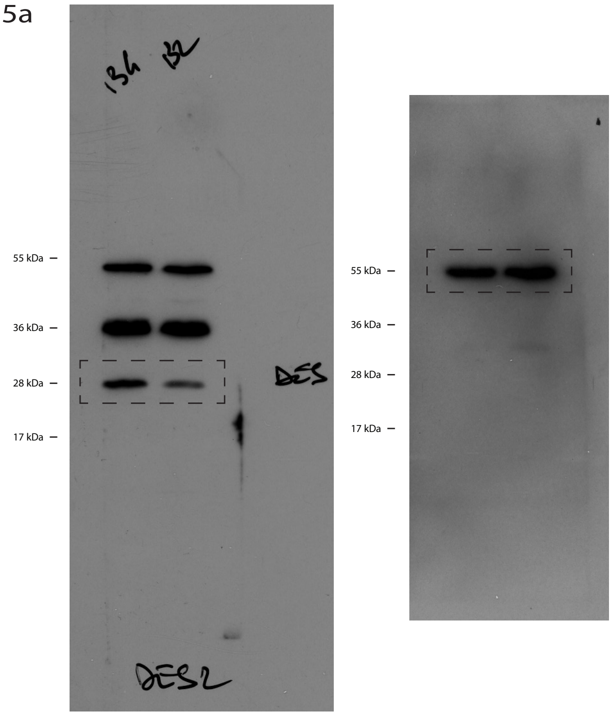

5b

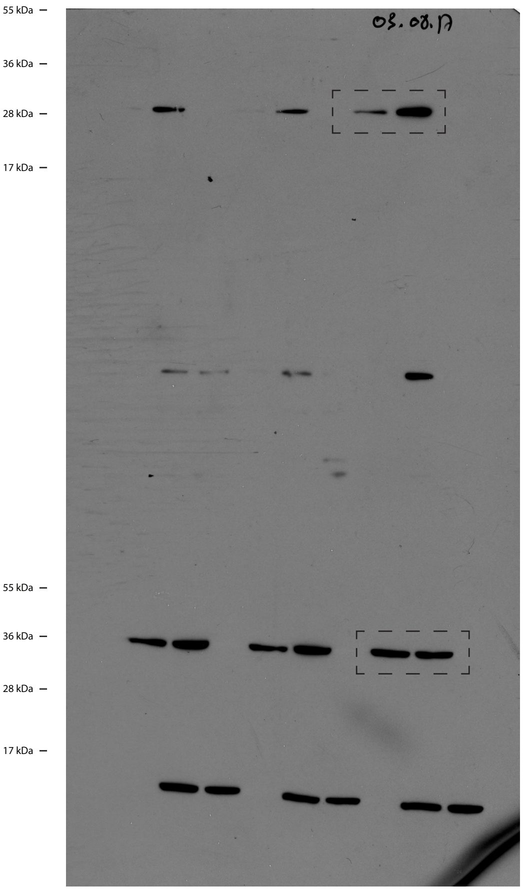

5c

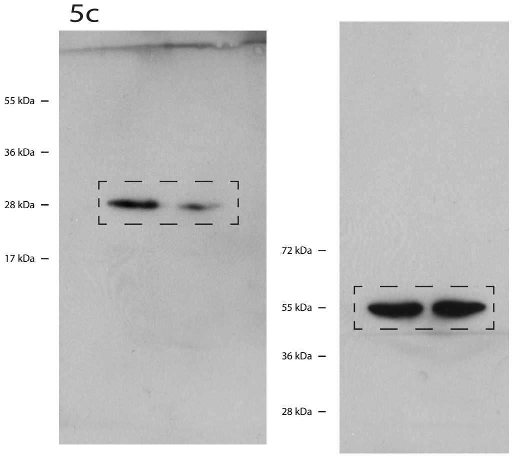

5d

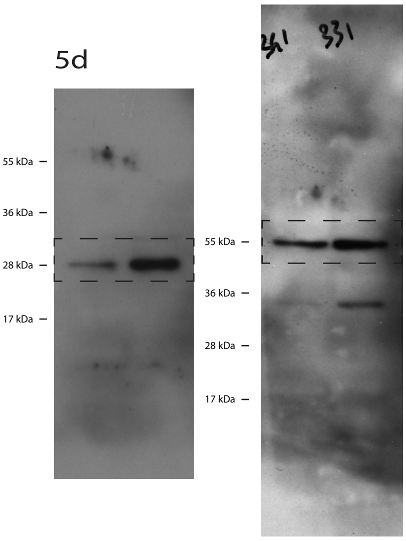

5j

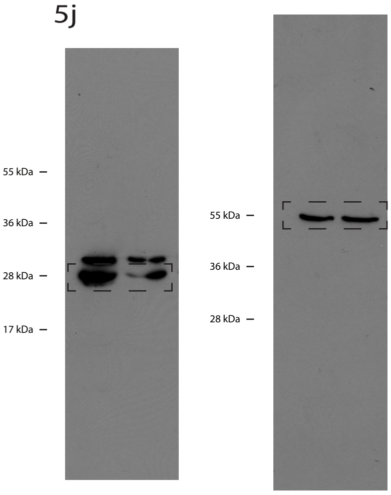

5l

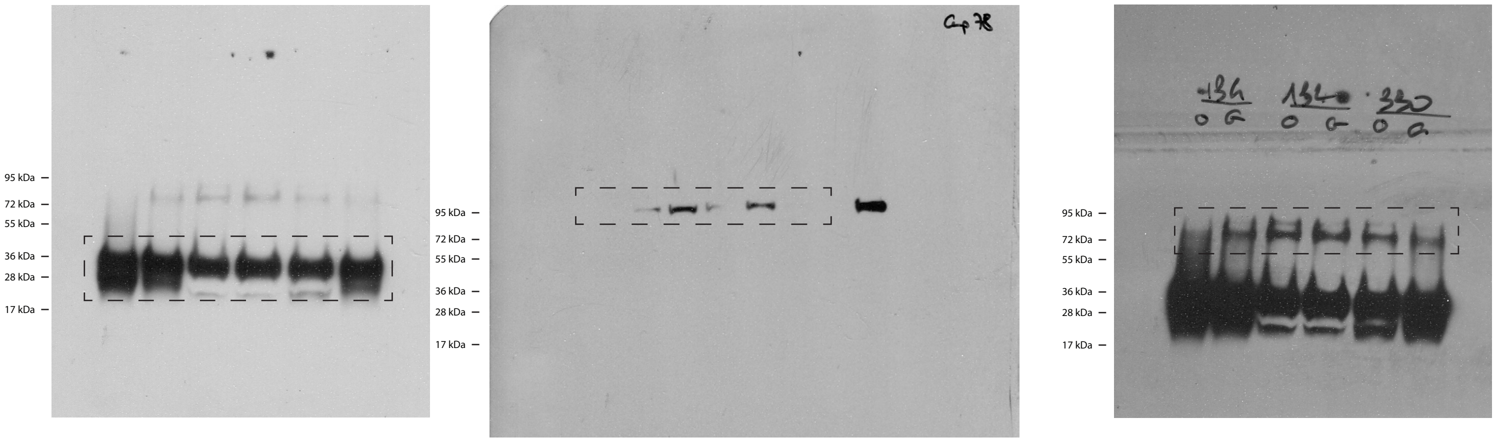

Supplementary information - original blots

6a

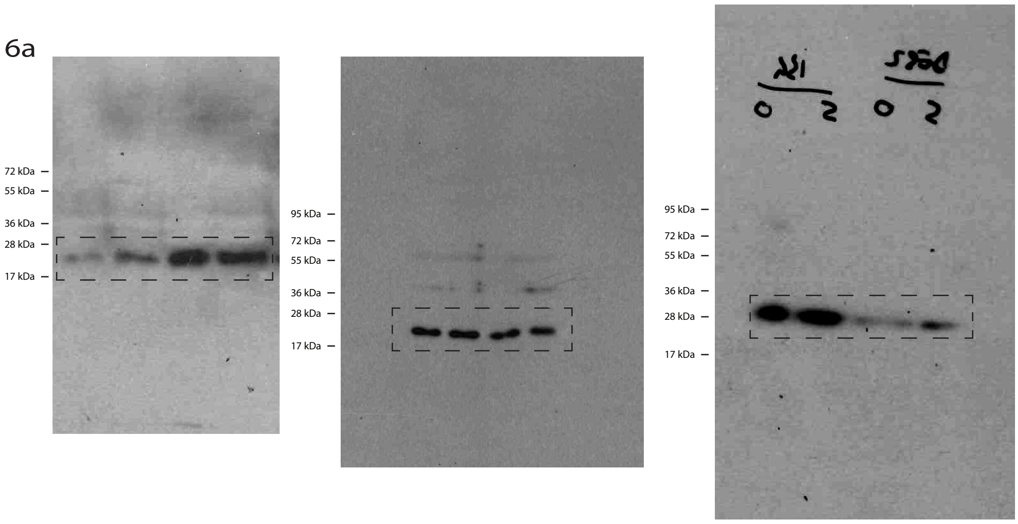

6b

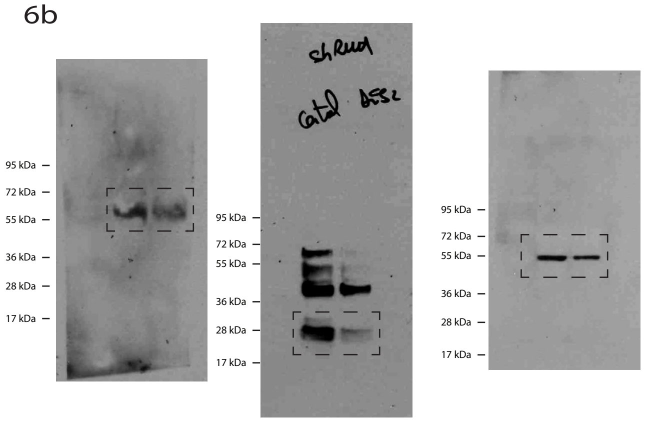

6c

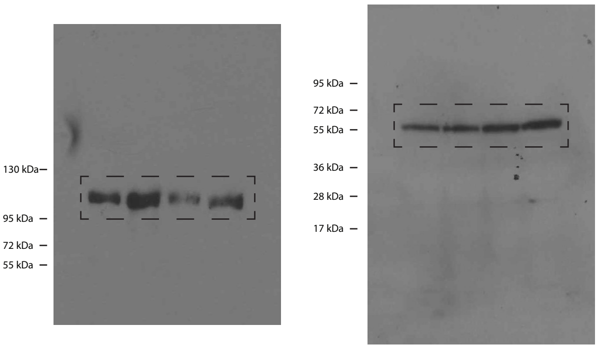

6d

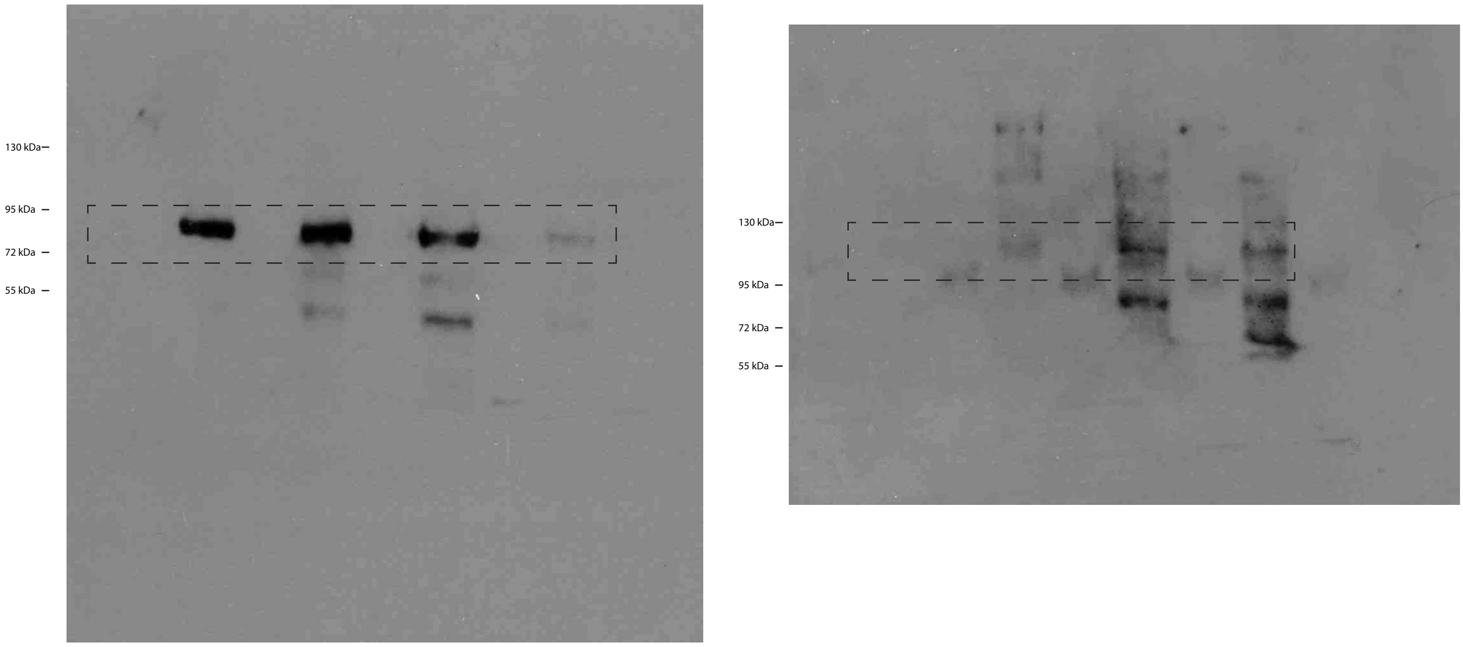

Supplementary information - original blots

7a

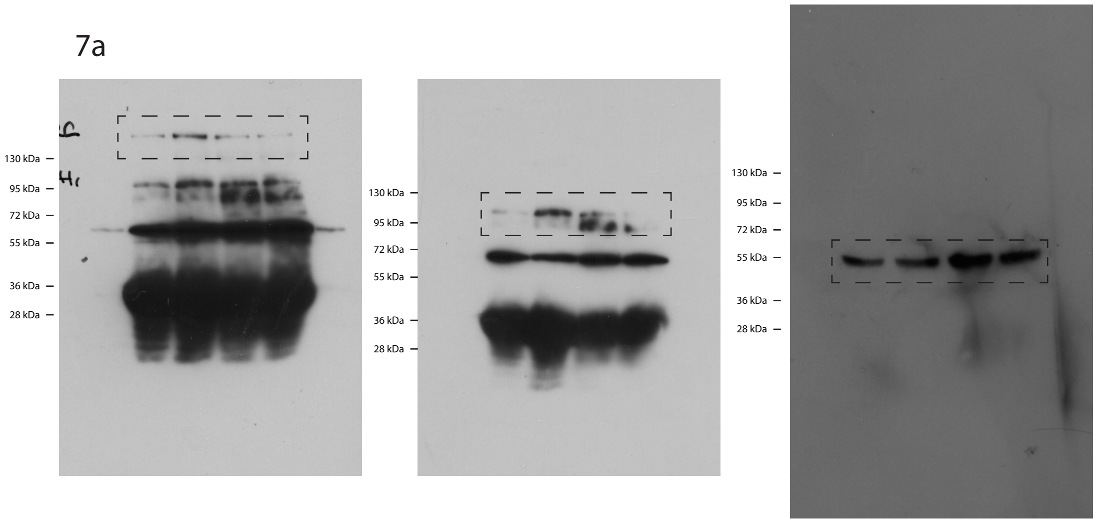

7b

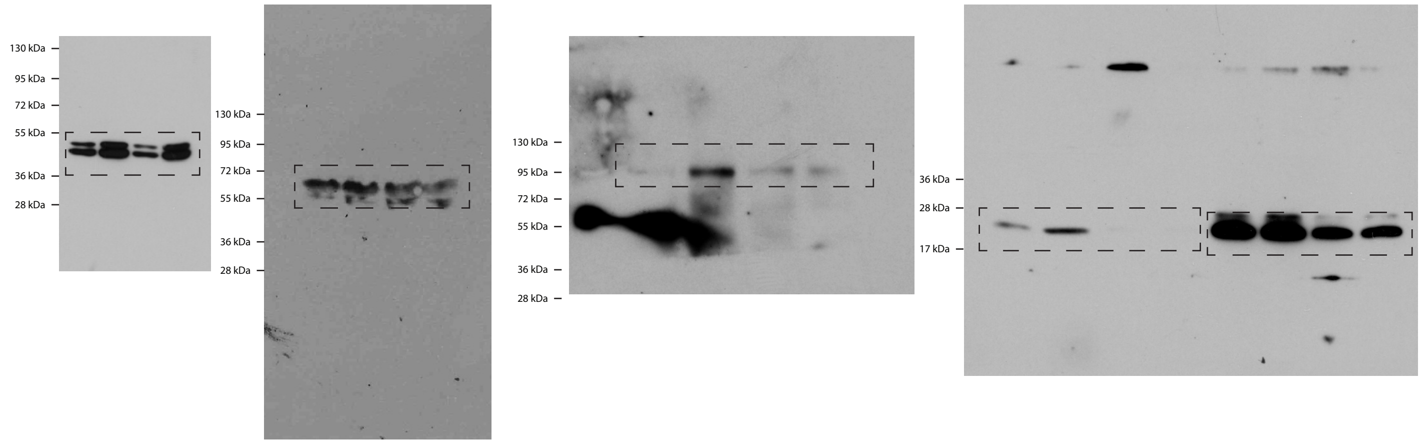

7b

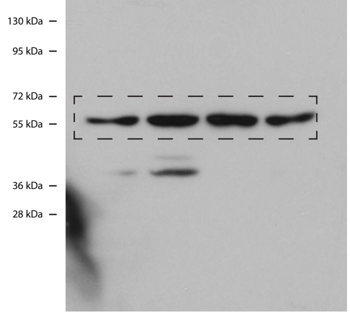

7c

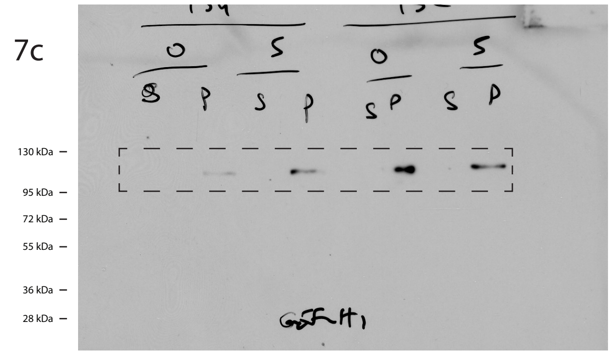

7c

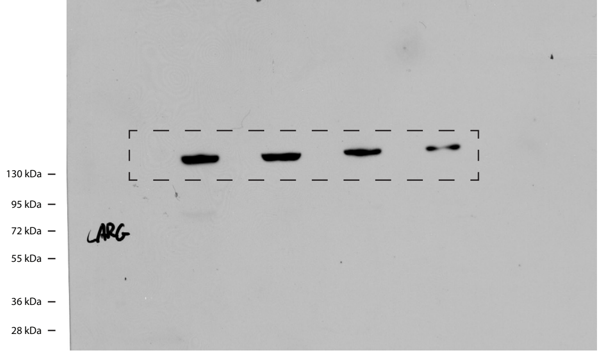

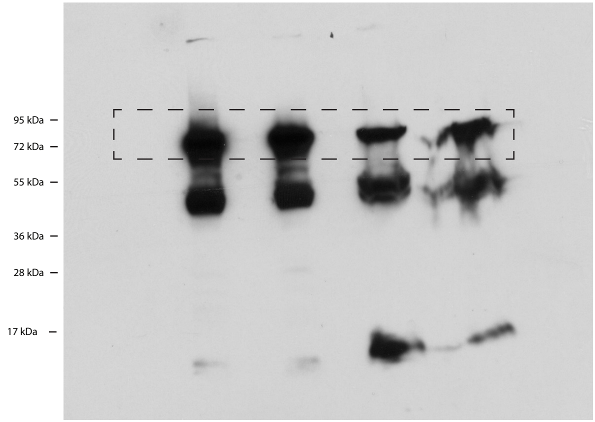

Supplementary information - original blots

7d

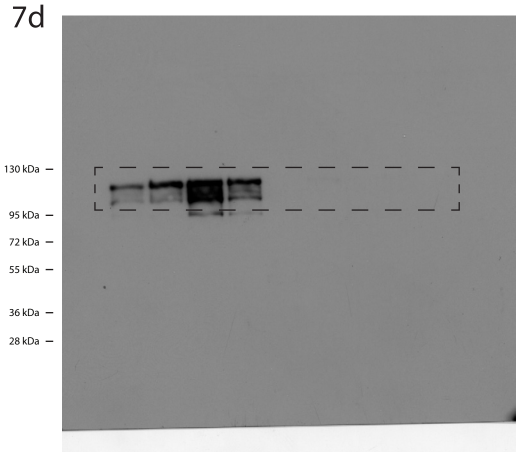

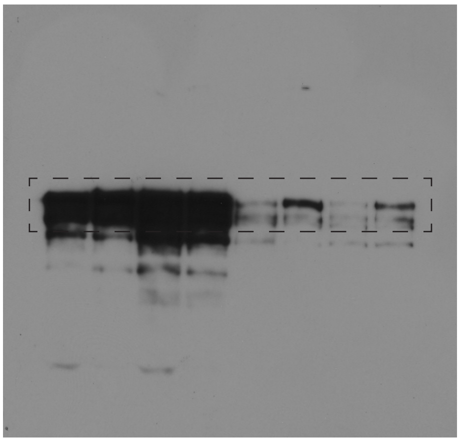

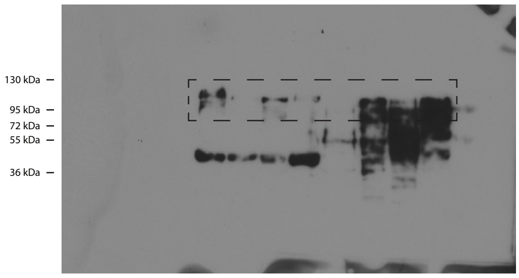

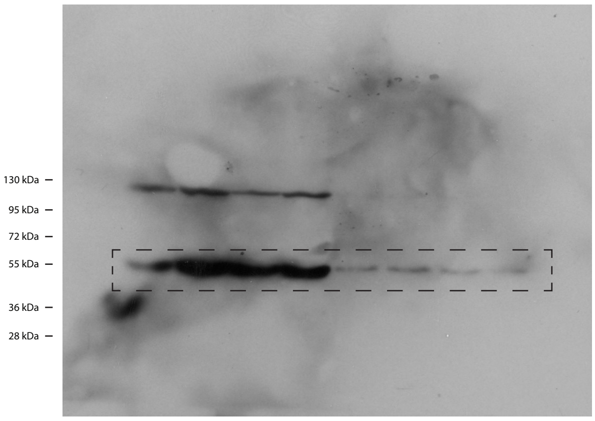

Supplementary information - original blots

8a

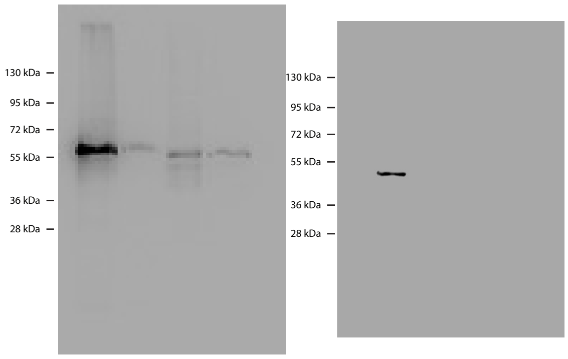

8b

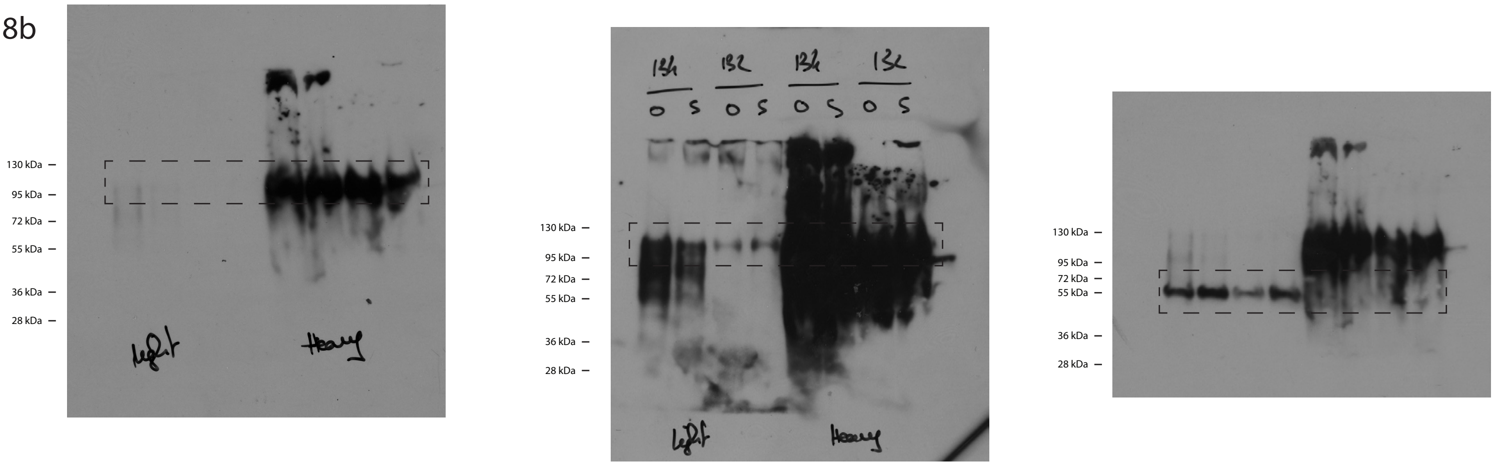

8d

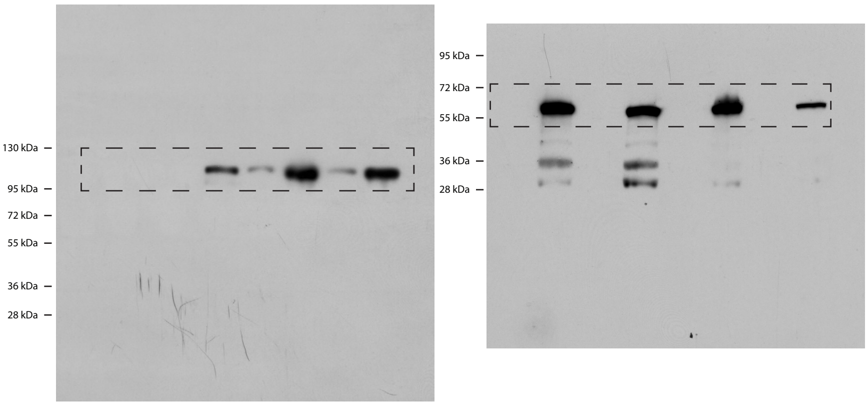

8e

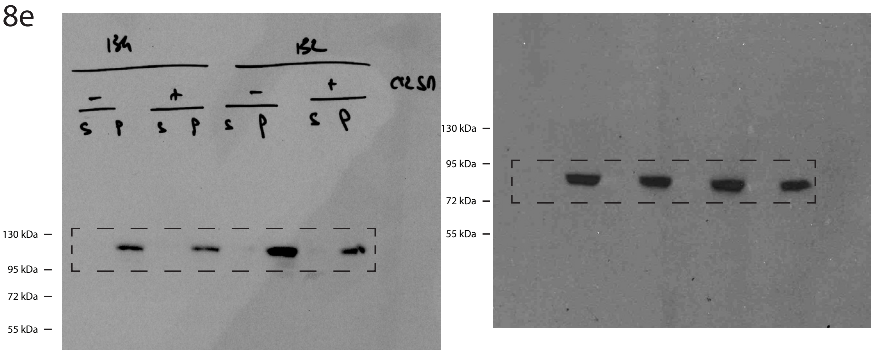

Supplementary References

1. Estévez, R. *et al.* The amino acid transport system y+ L/4F2hc is a heteromultimeric complex. *FASEB J.* **12,** 1319–1329 (1998).
2. Fenczik, C. A. *et al.* Distinct domains of CD98hc regulate integrins and amino acid transport. *J Biol Chem* **276,** 8746–52 (2001).
