# Supplementary material for: Cell metabolism regulates integrin mechanosensing via an SLC3A2-dependent sphingolipid biosynthesis pathway
Source: Nat Commun. 2018 Nov 19;9:4862. doi: 10.1038/s41467-018-07268-w (PMC6242995; doi:10.1038/s41467-018-07268-w)
Supplement: Supplementary file 4 — Description of Additional Supplementary Files [file 41467_2018_7268_MOESM4_ESM.docx]

Description of Additional Supplementary Files

**Supplementary Data 1:** Statistical analysis of the metabolomics dataset. Each metabolite from control, CD98hc null, C330S, C109S, C98T98E69 and C69T69E98 cells was compared to CD98hc re-repressing CD98hc null cells (after normalization). The table describes, for each metabolite, mean intensity, p-value in a Student’s t-test and Benjamini-Hochberg critical value at FDR 5%.
